# A Case of Diabetic Ketoacidosis-Induced Cardiomyopathy

**DOI:** 10.7759/cureus.83472

**Published:** 2025-05-04

**Authors:** Chelsea Crosby, Lei Gao

**Affiliations:** 1 Cardiology, Virginia Mason Franciscan Health Heart and Vascular, Burien, USA

**Keywords:** diabetic ketoacidosis, metabolic cardiomyopathy, st elevation, stress cardiomyopathy, takotsubo cardiomyopathy

## Abstract

Stress cardiomyopathy is a reversible syndrome that is usually triggered by emotional and physical stressors. We describe a patient who was admitted with diabetic ketoacidosis (DKA) and found to have metabolic-induced cardiomyopathy. The echocardiogram primarily revealed hypokinesis in the mid-segments, and left heart catheterization showed angiographically normal-appearing coronary vessels. The patient did not have significant emotional or physical stressors but did have a significant metabolic derangement. We suspect that the underlying mechanism of DKA-induced cardiomyopathy may differ from that of stress cardiomyopathy. Therefore, further observation and investigation are warranted.

## Introduction

Stress cardiomyopathy, also known as Takotsubo cardiomyopathy, is a reversible syndrome that is usually seen in postmenopausal women with associated chest discomfort and usually occurs after an episode of significant stress [1]. The stress is thought to cause a catecholamine-induced spasm versus toxicity of the myocardium [2]. Diabetic ketoacidosis (DKA) is a severe complication of diabetes and is known to cause metabolic acidosis and ketosis, and presents with abdominal pain, nausea, vomiting, lethargy, increased thirst, and increased urination [3]. The incidence of DKA per 10,000 admissions has increased over time from 32.04 in 2003 to 61.6 in 2017 [4]. To our knowledge, there have been only two incidences of DKA-induced stress cardiomyopathy in the literature [2,5]. The mechanism of this finding is not fully known, but some possible explanations include systemic inflammatory response syndrome (SIRS) coupled with pro-inflammatory cytokines that increase free radicals that cause myocardial stunning by inhibiting contractile proteins [6]. Another hypothesis is that severe acidemia causes proteolysis due to increased intracellular calcium [6]. In hopes of further acknowledging the presence of this type of case and the need for further vigilance, we would like to present a case of a 33-year-old female with metabolic cardiomyopathy induced secondary to DKA.

## Case presentation

**Figure 1:**
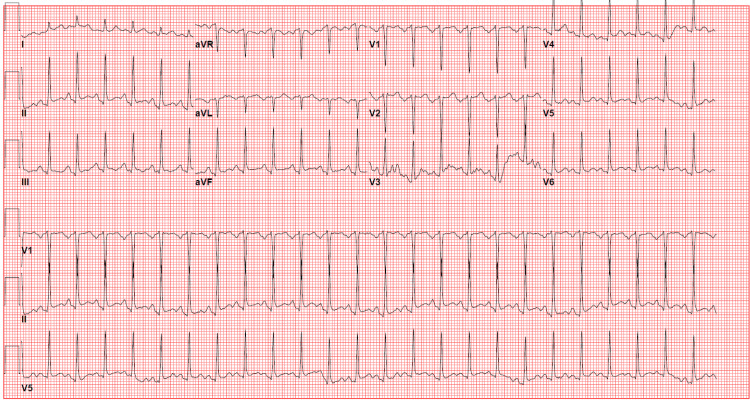
Initial 12-lead ECG. Normal sinus rhythm with non-specific ST-T wave changes. ECG: electrocardiogram

**Figure 2:**
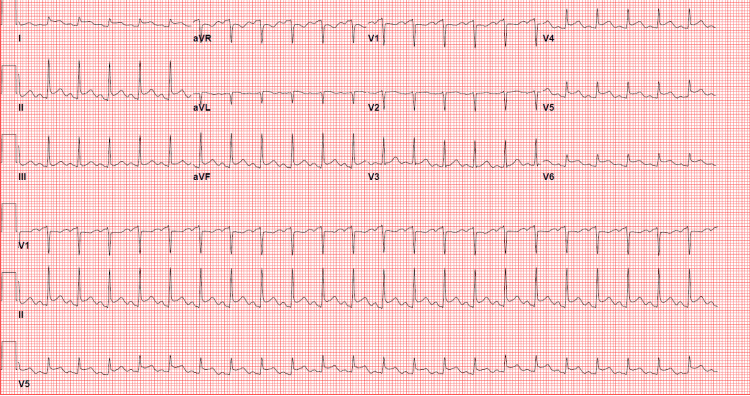
Subsequent 12-lead electrocardiogram showing sinus tachycardia with diffuse ST elevations.

A 33-year-old female with a past medical history of uncontrolled diabetes mellitus and hyperlipidemia presented to the emergency room after having nausea, vomiting, and poor oral intake for four days. The patient denied any chest pain, shortness of breath, or fever. She denied any personal history of cardiac disease but did have a father who had a myocardial infarction and stent in his forties. In the emergency room, the patient was found to be awake and alert with no abdominal wall tenderness. She was hypotensive (87/60) with a pulse of 92 beats per minute. The initial 12-lead electrocardiogram (ECG) showed sinus tachycardia with nonspecific ST-T wave changes (Figure 1). Initial blood work showed a serum glucose: 748 mg/dL (normal 65-99 mg/dL), serum potassium: 4.5 mmol/L (normal 98-111 mmol/L), serum bicarb: <5 mmol/L (normal 21-32 mmol/L), serum sodium: 128 mmol/L (normal 135-145 mmol/L, serum creatinine: 1.91 mg/dL (normal 0.5-1.2 mg/dL), and anion gap: 17 mmol/L (normal 2-11 mmol/Lk). The patient was acidotic with venous blood gas showing a pH of 6.887 and bicarbonate (HCO₃) of 4.9. Urinalysis showed 2+ ketones. The patient was diagnosed with DKA. Troponin I high-sensitivity (hs) was initially 23 pg/mL (normal <20 pg/mL). She was started on a normal saline bolus, sodium bicarbonate, and an insulin drip. A few hours later, the patient started to complain of substernal, non-radiating chest pain that was non-reproducible and described as a 5/10 pressure. ECG was then urgently repeated and showed sinus tachycardia with diffuse ST elevations, worse in lateral leads (Figure 2). The stat echocardiogram revealed a left ventricular ejection fraction of 40-45% with mild to moderate global hypokinesis of the left ventricle, mild to moderate mitral regurgitation with hypokinesis of mid-segments (Video 1-4). Venous blood gas at that time showed a pH of 7.160 with HCO₃ of 11.3. Troponin I hS trended up significantly and peaked at 20,085. The patient was then offered left heart catheterization but initially refused. She was also given IV Lasix for mild pulmonary edema. Goal-directed medical therapy was started with metoprolol succinate, losartan, and empagliflozin. On day two of admission, venous blood gas showed an improvement of pH to 7.346 with HCO₃ of 15.4.

**Video 1:** Initial echocardiogram parasternal long axis view.

**Video 2:** Initial echocardiogram parasternal short-axis view.

**Video 3:** Initial echocardiogram apical 4 chamber view.

**Video 4:** Initial echocardiogram apical 2 chamber view.

**Figure 3:**
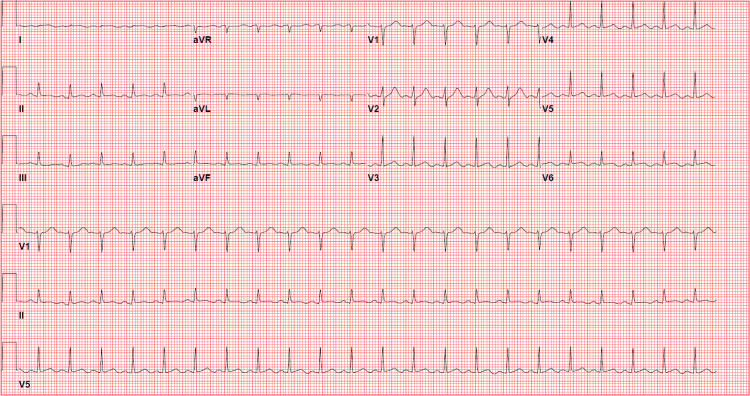
Repeat electrocardiogram on day four of admission after chest pain had resolved. Normal sinus rhythm with no significant ST-T wave changes.

**Figure 4:**
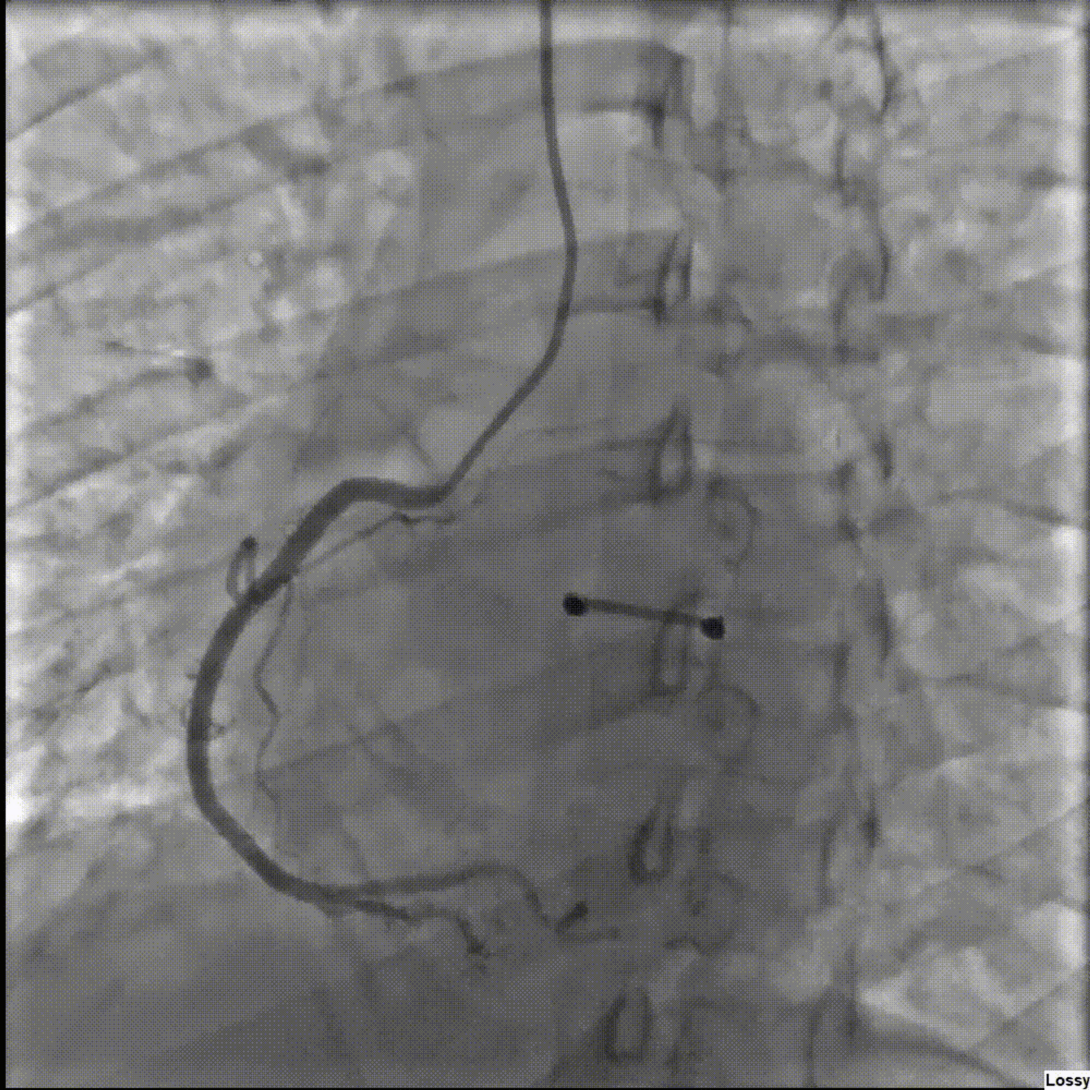
Left heart catheterization depicting no significant stenosis of the right coronary artery.

**Figure 5:**
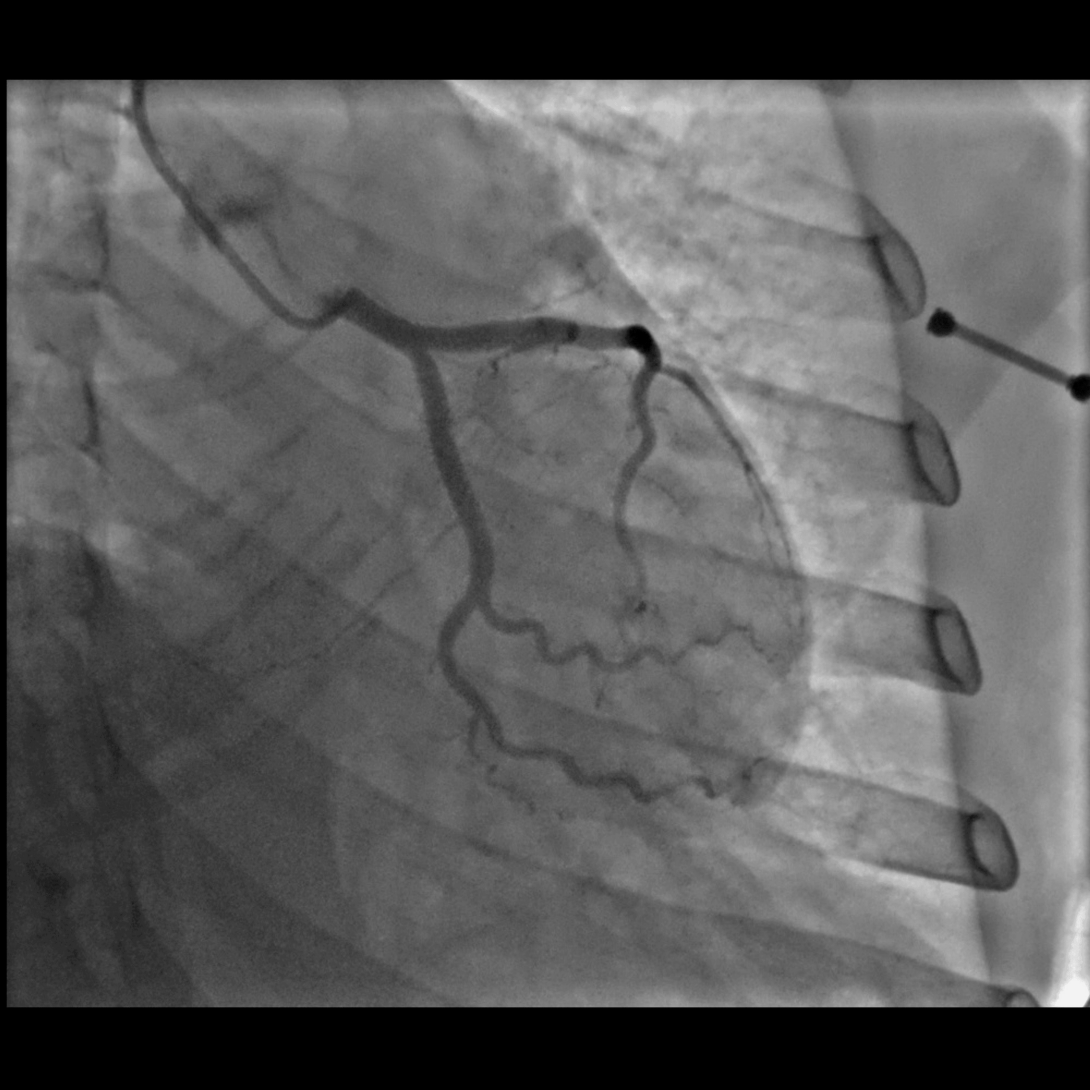
Left heart catheterization depicting no significant stenosis of left anterior descending and circumflex artery.

On day four of admission, she again had substernal, non-radiating chest pressure that resolved after a few hours. Repeat ECG showed ST elevations had resolved (Figure 3). The patient then agreed to left heart catheterization, which revealed angiographically normal appearing coronary arteries (Figures 4, 5). Echocardiogram was repeated on day seven of admission, which showed that the left ventricular ejection fraction had normalized to 55% with no further wall motion abnormalities (Videos 5-8). Her blood sugars were well controlled on empagliflozin. She was discharged home in stable medical condition. At a follow-up clinic visit 15 days after discharge, she was feeling well with no further chest pain or shortness of breath. Denied fever or chills. She continued on losartan and metoprolol and was recommended to maintain control of her diabetes. She was referred to her primary care physician for diabetes education and further diabetes management. 

**Video 5:** Subsequent echocardiogram parasternal long axis view.

**Video 6:** Subsequent echocardiogram parasternal short-axis view.

**Video 7:** Subsequent echocardiogram apical 4 chamber view.

**Video 8:** Subsequent echocardiogram apical 2 chamber view.

## Discussion

We wanted to present this case of DKA-induced cardiomyopathy with associated ST elevations, as only two other cases have been previously reported [2,5]. Stress cardiomyopathy was first described in Japan in 1990 [1]. This was previously thought to be a rare finding but is now found to be in 1-2% of patients with suspected acute coronary syndrome [1]. Fifteen to thirty cases per 100,000 people are seen in the US each year [1]. This is important as possible complications of stress cardiomyopathy can include heart failure, left ventricular outflow obstruction, and mitral regurgitation, which can all lead to cardiogenic shock, as did a case in 2023 [7]. Diabetes is thought to have a protective role in stress cardiomyopathy [8]. This case, however, shows that when presented with DKA, that protective role is less effective. It is unclear why DKA may cause cardiomyopathy. The pathophysiology of stress cardiomyopathy itself is unknown but is thought to possibly be secondary to catecholamine surge, causing cardiotoxicity and microvascular dysfunction [9]. The diagnosis is made based on the Revised Mayo Clinic Criteria [1]. The patient usually has transient changes to the left ventricular midsegments with or without apical involvement, no obstructive coronary artery disease on left heart catheterization, and new electrocardiographic changes or modest elevation in the troponin [1]. In this case, the echocardiogram primarily revealed hypokinesis in the mid-segments, ST elevations on ECG, and no obstructive coronary disease on left heart catheterization, which meets the listed criteria given by Mayo. The patient also had a dramatic rise in troponin from 23 pg/mL to 20,085 pg/mL, which also indicates stress cardiomyopathy based on the Mayo criteria. This patient did not have a significant stressor in her life but did have a significant metabolic derangement. We suspect that the underlying mechanism of DKA-induced cardiomyopathy may differ from that of stress cardiomyopathy. 

Other metabolic conditions besides DKA have also been found to cause cardiomyopathy. These include thyroid disorders such as Graves' disease, Hashimoto thyroiditis, and thyroid storm, as well as adrenal insufficiency, autoimmune polyendocrine syndrome, syndrome of inappropriate antidiuretic hormone secretion, and pheochromocytoma [10]. Another is the role of corticosteroid dysregulation as a cause of cardiomyopathy, both deficiency and exogenous steroids [11]. Further research is necessary to determine whether metabolic cardiomyopathy, including DKA-induced cardiomyopathy, shares the same underlying mechanisms. Another area of research could possibly be finding risk factors that would predispose a certain patient with DKA to have cardiomyopathy.

The significance of this case report is that it also serves as a wake-up call to the attention of DKA status. Not all cases of DKA lead to cardiomyopathy, raising questions about why some instances of DKA result in heart disease and what the prognosis is when DKA-related cardiomyopathy occurs. It is important that we stay vigilant for DKA patients with cardiac presentations that are atypical. We recommend at least checking an electrocardiogram for DKA patients and getting an echocardiogram if there are abnormal electrocardiogram findings. We are committed to continuing our investigation and will keep monitoring additional cases in search of answers.

## Conclusions

This case of DKA-induced cardiomyopathy shows the probable relationship of metabolic disorders and cardiomyopathy. This is valuable, as it is important to promptly recognize this as the cause of ST elevation and decreased ejection fraction to focus on treating the underlying disorder of DKA to lead to a more favorable prognosis. This is especially true in younger, female patients, as we reported here. 
